# Native Bacterial Communities of Two Italian Salso-Bromo-Jodic and Sulphurous Natural Mineral Waters

**DOI:** 10.3390/microorganisms13051038

**Published:** 2025-04-30

**Authors:** Angela Kuka, Irene Mileto, Marco Saler, Greta Petazzoni, Marta Corbella, Fausto Baldanti, Angela Faga, Giovanni Nicoletti

**Affiliations:** 1 Microbiology and Virology Unit, Fondazione IRCCS Policlinico San Matteo, 27100 Pavia, Italy; 2 Department of Clinical, Surgical, Diagnostic and Paediatric Sciences, University of Pavia, 27100 Pavia, Italy; 3 Advanced Technologies for Regenerative Medicine and Inductive Surgery Research Center, University of Pavia, 27100 Pavia, Italy; 4 Integrated Unit of Experimental Surgery, Advanced Microsurgery and Regenerative Medicine, University of Pavia, Via Adolfo Ferrata, 9, 27100 Pavia, Italy; 5 Surgery Unit, Azienda Socio-Sanitaria Territoriale di Pavia, 27100 Pavia, Italy

**Keywords:** bacterial communities, 16S, mineral water, sulphurous water, salso-bromo-jodic water, non-pathogenic microflora

## Abstract

A correlation between resident non-pathogenic bacterial populations in certain natural mineral waters and their beneficial effects has been established by several research groups. This study aims to characterize the bacterial composition of the Rivanazzano salso-bromo-jodic and sulphurous mineral waters (Pavia, Italy). Water samples were collected from natural sources and dispensing systems. DNA was extracted and subjected to 16S rRNA gene sequencing. Microbial composition, as well as alpha and beta diversity, were analyzed using amplicon sequence variants and compared across sampling sites. The predominant phyla in both waters were *Proteobacteria*, *Campylobacterota*, *Bacteroidota,* and *Desulfobacterota*. However, diversity at the family taxonomic level was recorded. In terms of bacterial diversity, waters collected from the dispensing systems within the spa resort were more similar between them than those from natural sources. The therapeutic properties of the Rivanazzano mineral waters are likely to be related to their combined mineral and biological composition.

## 1. Introduction

Natural mineral waters, whose therapeutic properties have been long recognised on empirical basis, are currently being investigated with rigorous experimental methods and have demonstrated antioxidant, wound-healing, skin hydration, and skin barrier recovery activities [1,2,3,4,5,6,7,8].

Over the last 15 years, our research group has been focusing on the regenerative properties of natural mineral waters. In detail, we have demonstrated this potential with human in vitro cell and ex vivo tissue culture experimental models both in calcium magnesium bicarbonate-based natural mineral water (Comano, Trento, Italy) [9,10,11,12,13,14] and in salso-bromo-jodic-based water (Rivanazzano, Pavia, Italy) [15].

Natural mineral waters are defined as bacteriologically pure, the latter definition meaning that they contain neither pathogenic microorganisms nor microorganisms indicating fecal or other contamination [16]. However, resident non-pathogen bacterial populations have been identified in some natural mineral waters, and a correlation between these bacterial microflora and the waters’ beneficial effects has been demonstrated. Indeed, *Aquaphilus dolomiae*, isolated from Avène thermal water (France), is able to regulate keratinocyte inflammatory and lymphocyte immune responses [17,18,19,20]. Similarly, *Vitreoscilla filiformis*, found in LaRoche-Posay thermal water (France), has been demonstrated to activate cutaneous regulatory T cells [21,22]. The native non-pathogenic microflora in Comano (Trento, Italy) natural mineral water display immune-modulating, anti-inflammatory, and even antibiotic effects [23].

A previous study of ours on both in vitro and ex vivo experimental models demonstrated some regenerative effects in a bacterial lysate from a new strain close to *Mesorhizobium erdmanii* (*Proteobacteria* phylum) [14], isolated in Comano mineral water.

Both favourable clinical effects and some experimental evidence [15,24] of Rivanazzano mineral waters are likely to be related to the combination of their well-known chemical–physical composition and their microbiological composition, which is still unknown to date. The aim of this experimental study was to characterize the native bacterial composition of Rivanazzano Terme (Pavia, Italy) salso-bromo-jodic and sulphurous mineral waters.

## 2. Materials and Methods

### 2.1. Thermal Bath Setting

Rivanazzano Terme (Pavia, Italy) is an Italian village located at 150 metres above sea level. The Rivanazzano Salus per Aquam (spa resort) has, unusually, two distinctly different water springs in the same site: a salso-bromo-jodic natural mineral water and a sulphurous one [25]. The chemical–physical composition of both waters is reported in Table 1.

The salso-bromo-jodic water is drawn up at a temperature of 13.9 °C from an approximately 350-metre-deep artesian well, consisting of a perforated 316 stainless steel “jacket” surrounded by draining gravel and a 316 stainless steel suction tube. A 316 stainless steel extraction pump is placed at a depth of roughly 60 m. The water is stored in a series of three underground 10,000 L settling tanks interconnected by 316 stainless steel pipes. The tanks are built in reinforced concrete covered with water-repellent plaster. After passing through the last tank, the water is fed through a long polyethylene pipeline until it reaches the spa resort 2.5 km away. Here, the water is shifted to two different storage tanks built in reinforced concrete covered with water-repellent plaster. One is filled with water at the native extraction temperature, and the other one is filled with water heated to 70 °C. The waters are then delivered to the individual bath tubs through a 316 stainless steel distribution network. In the bath tubs, the heated water is blended with water at the native temperature to provide adequate comfort for patients.

**Figure 1:**
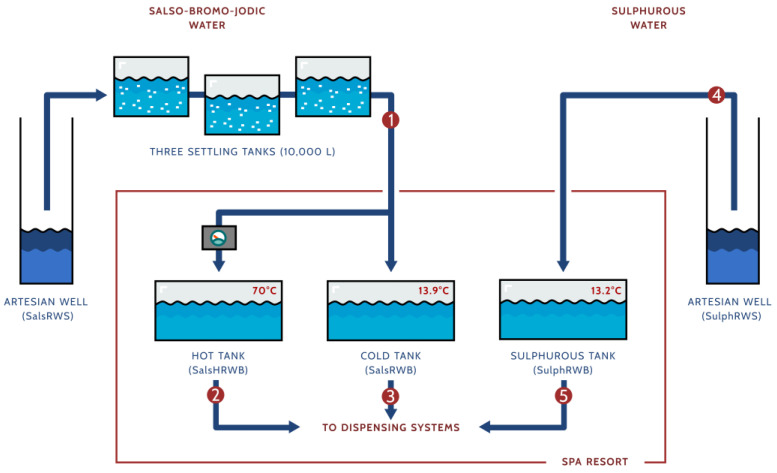
Schematic representation of the Rivanazzano Terme water network. Collection sites are indicated by numbers in red circles: (1) salso-bromo-jodic water from the natural source collected at the exit of the last settling tank (SalsRWS); (2) heated salso-bromo-jodic water collected at the exit of its storage tank within the spa resort (SalsHRWB); (3) salso-bromo-jodic water at the natural source temperature collected at the exit of its storage tank within the spa resort (SalsRWB); (4) sulphurous water collected at the natural source (SulphRWS); and (5) sulphurous water collected at the exit of its storage tank within the spa resort (SulphRWB).

The sulphurous water is extracted at 13.2 °C from an approximately 12-metre-deep artesian well with the same structural features as the salso-bromo-jodic water well. The water is delivered to an underground storage fiberglass tank within the spa resort through a dedicated polyethylene pipeline, which is almost 2 km long. Through a 316 stainless steel pipeline, the sulphurous water is then delivered to inhalation dispensing systems and to a single individual bath tub, where it is extemporaneously warmed according to the patient’s requirements (Figure 1).

The polyethylene pipelines from the natural sources to the spa resort are regularly cleaned with a reverse-flow rinse with tap water on a 3-monthly basis. The storage tanks in the spa resort are maintained every year with sediment removal and tap water rinsing. The stainless steel pipelines from the storage tanks to the different dispensing systems and to the individual bath tubs in the spa resort are rinsed with reverse-flow tap water for 90 min every night.

### 2.2. Study Design

#### 2.2.1. Samples Collection

The water samples were collected at three different time points: May, June, and July 2022. Waters were collected from five sample sites to obtain a comprehensive overview of the bacterial communities (Figure 1):

1. The exit of the third settling tank at the site of the salso-bromo-jodic natural source (SalsRWS).
2. The exit of the salso-bromo-jodic storage tank at 70 °C within the spa resort (Hot tank; SalsHRWB).
3. The exit of the salso-bromo-jodic storage tank at the natural source temperature within the spa resort (Cold tank; SalsRWB).
4. The sulphurous natural source (SulphRWS).
5. The exit of the sulphurous storage tank at the natural source temperature within the spa resort (Sulphurous tank; SulphRWB).

For each site and collection period, water samples were gathered by a single individual using an aseptic procedure, as reported in a previous study [10]. In each site, water samples were collected in two 2-litre sterile pots. Then, they underwent centrifugation to remove macroscopic sediment. Filtration was performed using cellulose filters with a porosity of 0.1 µm (Whatman, GE HealthCare Life Sciences, Chicago, IL, USA), and filters were vortexed in an aliquot of water before starting with sample processing. Each sample was then concentrated in 2 mL tubes to proceed with the genomic DNA extraction. Two technical replicates were carried out for each water sample.

#### 2.2.2. Library Preparation and Sequencing

Bacterial DNA was extracted using NucleoSpin Tissue (Macherey–Nagel^®^, Düren, Germany) following the manufacturer’s instructions, and the rRNA 16S region was sequenced by BMR Genomics s.r.l. (Padova, Italy). The V3 and V4 hypervariable regions were targeted using Pro341F (5′-CCTACGGGNBGCASCAG-3′) and Pro805R (Rev 5′-GACTACNVGGGTATCTAATCC-3′) primers for Bacteria and Archaea [26], modified with Illumina sequencing tails (5′-TCGTCGGCAGCGTCAGATGTGTATAAGAGACAGCCTACGGGNBGCASCAG-3′ and Rev 5′-GTCTCGTGGGCTCGGAGATGTGTATAAGAGACAGGACTACNVGGGTATCTAATCC-3′, respectively) and sequenced with a paired-end set up (2 × 300 bp, Illumina, San Diego, CA, USA).

#### 2.2.3. Bioinformatic Analysis

The raw sequence data were processed in QIIME2 (vr. 2022.8) [27] using the DADA2 algorithm [28] to trim reads (last 5 nt for forward and 30 nt for reverse reads), remove low-quality reads, denoise, estimate run-specific error rates, and infer amplicon sequence variants (ASVs). Within the algorithm, quality control included chimaera detection and removal, sequence error elimination, and singleton exclusion. Taxonomy was assigned on ASVs training a naive Bayes classifier with the SILVA 138 database [29]. Multiple sequence alignments were performed using MAFFT (vr. 7.505) [30] and FastTree 2 (vr. 2.1.11) [31] was used to infer a rooted phylogenetic tree.

#### 2.2.4. Diversity Analyses

The ⍺-diversity metrics (i.e., richness and Shannon diversity index) of bacterial communities were computed at all taxonomic levels on even rarefied data to analyse the within-site diversity using the phyloseq package (version 1.40.0) [32]. The β-diversity was computed at all taxonomic levels. First, it was computed to assess the diversity within sites for the three different water sample collection times using UniFrac weighted distances and then to compare sampling sites using Bray–Curtis distances on even rarefied data (three from salso-bromo-jodic water and two from sulphurous water). Principal coordinate analysis (PCoA) was used for visualisation, and a permutational multivariate analysis of variance (PERMANOVA) was performed with 999 permutations on distances using the vegan R package (version 2.6-4) [33]. Once the homogeneity between time points within the same site had been assessed, data were pooled by the median of the ASV count to evaluate the bacterial composition. To compare relative abundance distributions between sites in salso-bromo-jodic water and sulphurous water, we used the Mann–Whitney U-test corrected with the Bonferroni method in the case of the first water type. Statistical significance was assumed with a *p*-value < 0.05.

## 3. Results

### 3.1. Bacterial Community Structure of the Waters

The filtering raw sequence quality process retained a total of 287,570 sequences in the final dataset. Each sample was represented by a range of between 4877 and 17,048 high-quality sequences, which were assigned to 1114 ASVs across 30 water samples. Analysis of β-diversity within sites at three different time points revealed no statistically significant differences at any taxonomic level. Therefore, the samples from three time points were gathered to compare the bacterial composition of the different sites for each water type.

#### 3.1.1. Bacterial Community Structure in Salso-Bromo-Jodic Waters

**Figure 2:**
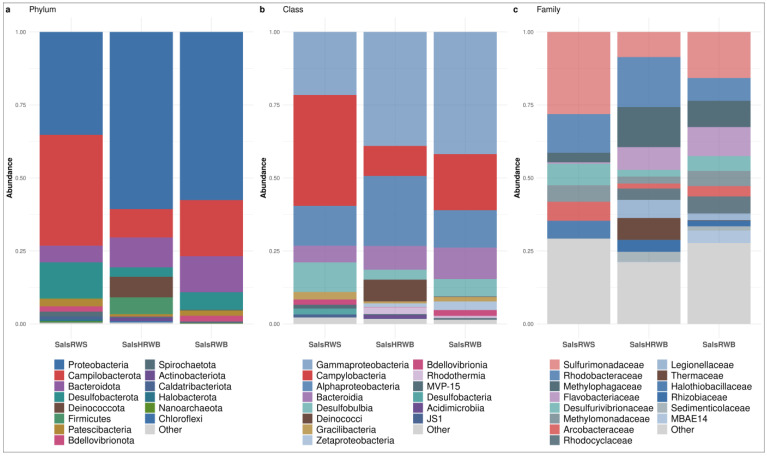
Most representative taxa at the (**a**) phylum, (**b**) class, and (**c**) family level of bacterial communities in salso-bromo-jodic waters divided by sample site: salso-bromo-jodic water collected at the natural source (SalsRWS); heated salso-bromo-jodic water (SalsHRWB); and salso-bromo-jodic water at natural source temperature (SalsRWB) collected at the exit of the respective storage tanks within the spa resort. For the barplots, samples of each site were pooled by median ASV count.

The most representative phyla detected in salso-bromo-jodic waters were *Proteobacteria* (57.57%; IQR: 12.70%), *Campylobacterota* (19.21%; IQR: 14.12%), and *Bacteroidota* (10.23%; IQR: 3.32%), with traces of *Desulfobacterota* (6.06%; IQR: 4.49%). The top four phyla accounted for 93.07% of the total ASVs. Interestingly, larger amounts of *Proteobacteria* were found in both the water at natural source temperature and the heated water samples collected within the spa resort (57.57% and 60.65%, respectively), compared to the samples collected at the natural source (35.25%; *p*-value < 0.05). With *Bacteroidota*, a statistical difference was observed in the samples collected at the natural source vs. the ones at the spa resort at natural source temperature (5.70% and 10.23% respectively; *p*-value < 0.05). Conversely, no statistically significant difference was detected comparing the samples collected at the natural source vs. the heated ones at the spa resort (12.34%). *Campylobacterota* and *Desulfobacterota* were the most prevalent in the water collected at the natural source (37.96% and 12.20%, respectively), vs. the heated water (19.21% and 6.06%) and at the natural source temperature (9.73% and 3.22%) within the spa resort. Other phyla were *Firmicutes* and *Deinococcota*. These were detected exclusively in heated water (5.78% and 7.03%, respectively) (Figure 2a).

The *Proteobacteria* phylum includes *Gammaproteobacteria* (39.03%; IQR: 10.81%), *Alphaproteobacteria* (13.65%; IQR: 7.94%), and *Zetaproteobacteria* (1.23%; IQR: 1.91%) classes. *Gammaproteobacteria* was the most representative class in the waters collected at spa resort, both at the natural temperature (41.83%) and after heating to 70 °C (39.03%). However, in the samples collected at the natural source, this was not the case (21.60%; *p*-value < 0.05). *Alphaproteobacteria* and *Zetaproteobacteria* displayed a higher prevalence in the waters collected at the spa resort, both after heating (23.96% and 1.23%, respectively) and at the natural source temperature (12.80% and 2.95%, respectively) vs. the water collected at the natural spring source (13.65% and 0.00%, respectively; Figure 2b).

Regarding *Gammaproteobacteria*, the *Methylomonadaceae* presented an equal abundance both in the waters collected at the natural source (5.63%) and the ones at the spa resort at the natural source temperature (5.17%), while a lesser percentage was found in the heated water (2.39%).

Furthermore, *Halothiobacillaceae*, and *Rhodanobacteraceae* were relatively plentiful in the water at the natural source (6.10% and 2.08%, respectively), while only traces were observed in the waters collected at the other sites (*p*-value < 0.05 for the *Halothiobacillaceae*). Conversely, *Rhodocyclaceae*, *Legionellaceae*, *Sedimenticolaceae*, *Gallionellaceae*, and MBAE-14 were found in the waters collected at the spa resort, both after heating (3.86%, 6.22%, 3.45%, 0.94% and 0.14%, respectively) and at the natural source temperature (5.84%, 2.31%, 1.51%, 3.13% and 4.22%, respectively), while the aforementioned taxa were absent from water at the natural source.

Moreover, the *Methylophagaceae* family was prevalent in the heated water (13.72%), vs. the water at the natural source temperature collected at the spa resort (9.01%) and at the natural source (3.26%). A higher abundance of *Methylococcaceae* and *Vibrionaceae* (4.68% and 1.47%, respectively) was also observed in heated water. In the water collected at the spa resort at the natural source temperature, *Pseudomonadaceae* and *Alteromonadaceae* (2.12% and 2.82%) were the most represented families compared to at the other water collection sites (Figure 2c).

*Alphaproteobacteria* were well represented in the heated water. As for the abundance of the *Rhodobacteraceae* family (17.11%), it was significantly higher in this water vs. the water collected at the spa resort at the natural source temperature (7.81%, *p*-value < 0.05). However, its abundance was not statistically significant in the water collected at the natural source (13.26%). Moreover, the *Defluviicoccales* (1.28%) and *Rhizobiaceae* (4.00%) families were found only in the heated water. Finally, the *Zetaproteobacteria* class was represented mainly by the *Mariprofundaceae* family, which was detected only in the waters collected at the spa resort, both at the natural source temperature (2.97%) and after heating at 70 °C (1.24%). This family was absent from the water collected at the source (Figure 2c).

Concerning the *Campylobacterota* phylum, the *Campylobacteria* was the most abundant class in the water from the natural source (37.96%) vs. the waters collected at the spa resort, both after heating (19.21%) and at the natural source temperature (10.30%; Figure 2b). Within the *Campylobacterales* order, *Sulfurimonadaceae* accounted for 28.13% of total family abundance in the water from the natural source but only for 15.77% and 8.62% in the waters collected at the spa resort after heating and at the natural source temperature, respectively. Other *Campylobacterales*, such as *Arcobacteraceae* and *Sulfurospirillaceae*, were also prevalent in the water at the natural source (6.53% and 3.31%, respectively), vs. the waters collected at the other sites (Figure 2c).

The *Bacteroidota* phylum was mainly represented by *Bacteroidia* and *Rhodothermia* classes (Figure 2b). Within the *Bacteroidia* class, the *Bacteroidetes* VC2.1 Bac22 accounted for 3.90% of the total family abundance in the water from the natural source, while only a few traces were detected in the other water collection sites (0.68% at the spa resort at the natural source temperature and 0.30% in the heated water, *p*-value < 0.05). Furthermore, the *Flavobacteriaceae* family was particularly abundant in the water collected at the spa resort at the natural source temperature (9.92%) vs. the heated water (7.80%) and the water from the natural source (0.49%, *p*-value < 0.05). Finally, the *Rhodothermaceae* family, part of the *Rhodothermia* class, was mainly represented in the heated water (2.33%), vs. the water from the natural source and water collected at the spa resort at the natural source temperature (0.00% and 0.76%, respectively; Figure 2c).

*Desulfobulbia* (*Desulfobacterota* phylum) was the most abundant class in the water from the natural source (10.12%) vs. the water collected at the spa resort, both at the natural source temperature (6.02%) and after heating at 70 °C (3.40%; Figure 2b). This was confirmed for the *Desulfobulbales* order too, whose *Desulfurivibrionaceae* and *Desulfocapsaceae* families accounted for 7.38% and 2.74%, respectively, of the total class abundance in the water from the natural source (Figure 2c).

Finally, the *Deinococci* class (*Deinococcota* phylum) was more represented in the heated water (7.44%) vs. the water collected at the other sites, in which only a few traces were observed (*p*-value < 0.05). At the family taxonomic level, *Thermaceae* accounted for 7.48% of the total family abundance in the heated water (Figure 2c).

#### 3.1.2. Bacterial Community Structure in Sulphurous Waters

**Figure 3:**
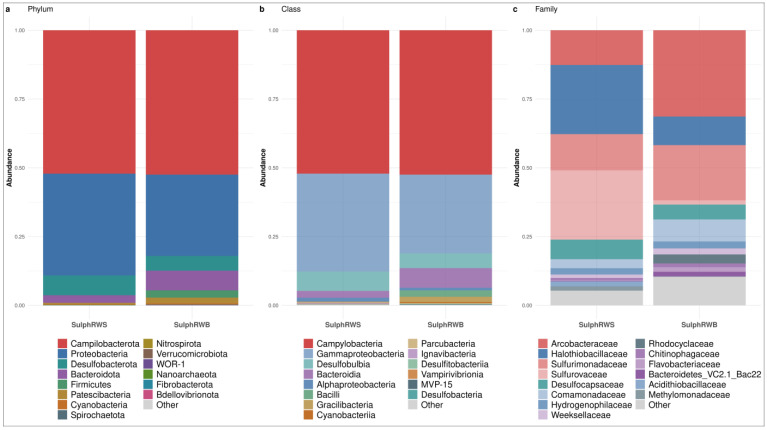
Most representative taxa at the (**a**) phylum, (**b**) class, and (**c**) family level of communities in sulphurous waters divided by sample site: sulphurous water collected at natural source (SulphRWS) and sulphurous water collected at the natural source temperature (SulphRWB) within the spa resort. For the barplots, samples of each site were pooled by median ASV count.

The most representative phyla in sulphurous waters were *Campylobacterota* (52.30%; IQR: 0.17%), *Proteobacteria* (33.28%; IQR: 3.72%), *Desulfobacterota* (6.27%; IQR: 0.91%), and traces of *Bacteroidota* (4.96%; IQR: 2.20%). As for *Firmicutes*, these were only observed in the sulphurous water collected at the spa resort (1.32%, *p* < 0.05; Figure 3a).

All *Campylobacterota* were assigned to the *Campylobacteria* class. In the water from the natural source, most *Campylobacterales* were detailed in *Sulfurovaceae* (25.18%) vs. the sulphurous water collected at the spa resort where they accounted for only 1.52% (*p* < 0.05%). Other *Campylobacterales* were represented by *Sulfurimonadaceae* (13.18% at the spring and 20.07% at the spa resort) and *Arcobacteraceae* (12.63% at the spring and 31.37% at the spa resort, *p* < 0.05) (Figure 3b,c).

As for *Proteobacteria*, they were mainly composed of *Gammaproteobacteria* (32.10%; IQR: 3.47%; Figure 3b). The prevalence of *Halothiobacillaceae* was higher in the water collected at the source (25.11%) vs. that collected at the spa resort (10.39%, *p*-value < 0.05); this was also noticed for *Acidithiobacillaceae* and *Methylomonadaceae* (1.83% and 1.53% at the spring). Conversely, *Comamonadaceae*, *Rhodocyclaceae* and *Sulfuricellaceae* were most prevalent in the water collected at the spa resort (8.01%, 3.29% and 1.48%, respectively). *Hydrogenophilaceae*, instead, were equally abundant at both sites (2.54% in the water at the spa resort and 2.30% at the source; Figure 3c).

The *Desulfobacterota* phylum was represented only by the *Desulfobulbia* class. The *Desulfocapsaceae* family was more prevalent in the water at the source (7.10%) vs. that at the spa resort (5.40%; Figure 3b,c).

The *Bacteroidota* phylum was mainly represented by the *Bacteroidia* class, and it was particularly abundant in the water collected at the spa resort. In particular, the most represented families were *Weeksellaceae* (2.17%), *Bacteroidetes* VC2.1 Bac22 (Candidatus *Sulfidibacteriales*, 1.71%), *Flavobacteriaceae* (1.65%), and *Chitinophagaceae* (1.37%; Figure 3b,c).

Finally, the *Firmicutes* phylum was mainly represented by *Erysipelatoclostridiaceae*. This was observed only in the water collected at the spa resort, with a prevalence of 1.66%.

### 3.2. Diversity Analyses

Alpha and beta diversity were computed to compare diversity in different water types.

#### 3.2.1. Diversity Analysis in Salso-Bromo-Jodic Water

**Figure 4:**
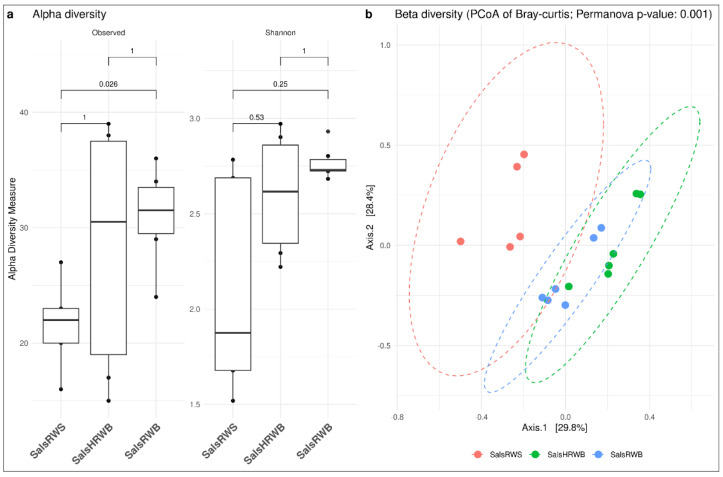
Diversity analyses. (**a**) Alpha and (**b**) Beta diversity at the family taxonomic level for salso-bromo-jodic waters at the different collection sites: salso-bromo-jodic water collected at the natural source (SalsRWS); heated salso-bromo-jodic water (SalsHRWB); and salso-bromo-jodic water at natural source temperature (SalsRWB) collected at the exit of the respective storage tanks within the spa resort.

Within the salso-bromo-jodic water, at the family taxonomic level, a more diverse community was observed, based on richness, in the water collected at the spa resort at the natural source temperature vs. the water collected at the source (*p* < 0.05) and also the heated water, even though no significant difference was observed for the latter comparison. The Shannon index was higher for the salso-bromo-jodic water collected at the spa resort at the natural source temperature (median: 2.73%; IQR: 0.06%) and the heated one (median: 2.62%; IQR: 0.51%) when compared to the water from the source (median: 1.88%; IQR: 1.01%). Regarding beta diversity, a diversification in the microbial community was observed at the different sites of water collection (*p* < 0.05); from the PcOA, more homogeneity was recorded between the waters collected at the spa resort both at the natural source temperature and after heating at 70 °C (Figure 4).

#### 3.2.2. Diversity Analysis in Sulphurous Water

**Figure 5:**
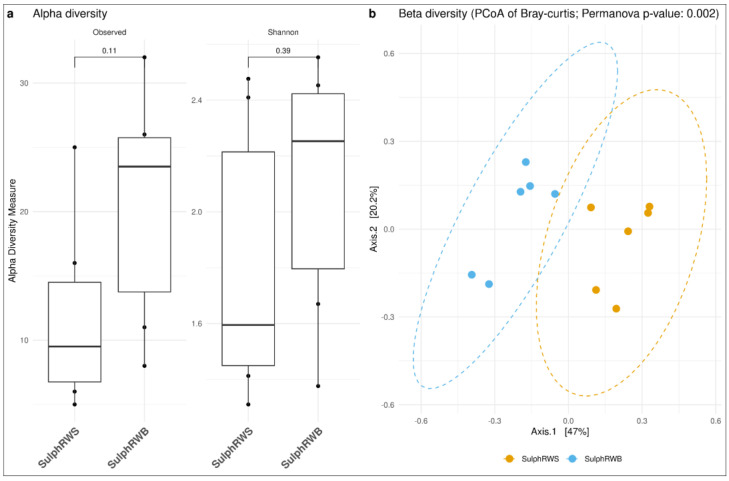
Diversity analyses. (**a**) Alpha and (**b**) Beta diversity at the family taxonomic level for sulphurous waters divided by sample site: sulphurous water collected at natural source (SulphRWS) and sulphurous water collected at the natural source temperature (SulphRWB) within the spa resort.

In the sulphurous waters, greater diversity was found in the water collected at the spa resort vs. that from the source. Indeed, the Shannon index was 2.25 (IQR: 0.63%) for the spa resort and 1.59 (IQR: 0.77%) for the source. This diversity also expressed itself in the beta diversity, where a statistically significant difference was recorded between the two sites (Figure 5).

## 4. Discussion

The sequential sampling of both Rivanazzano salso-bromo-jodic and sulphurous mineral waters revealed a constant and specific microbiota composition for each water type. The bacterial populations observed are typical of deep-water sources in a marine-derived geological environment. In fact, the Rivanazzano spa resort lies within Oltrepò Pavese (Pavia, Italy), in the foothills of the northernmost portion of the Apennine range. It was formed on the bottom of the Ligurian–Piedmontese oceanic basin and is made up of vast surfaces of sedimentary blankets of ancient alluvial origin (Pleistocene era) and more recent marine origin (Miocene–Upper Pliocene era). The geology of this territory includes a gypsum–sulphur formation formed at the end of the Miocene during the “Messinian salinity crisis” and oil-related deposits which usually accompany the salso-bromo-jodic springs [34].

At the natural sources, the salso-bromo-jodic and sulphurous waters show marked differences in mineralization and composition. While both have similar natural source temperatures (13.9 °C vs. 13.2 °C), the salso-bromo-jodic water exhibits significantly higher conductivity (8100 μS/cm) and fixed residue (12,400 mg/L), indicating greater total dissolved solids. Chemically, the salso-bromo-jodic water is rich in Na^+^ (4490 mg/L), Cl^−^ (7300 mg/L), Mg^2+^ (78.5 mg/L), and Sr^2+^ (15.8 mg/L). In contrast, sulphurous water is characterized by its high H_2_S concentration (11.9 mg/L)—which is absent from the salso-bromo-jodic type—and higher SO_4_^2−^ content (283 mg/L vs. 35.7 mg/L).

Actually, the natural mineral waters’ favourable effects are traditionally attributed to their chemical–physical composition, which is unique to every spring and specifically active on the skin [7,8,15,35], even though, for 20 years now, synergy with some specific non-pathogenic bacterial populations has been recognized [4,5,13,14,17,18,19,20,21,22,36]. We believe that, like other natural mineral waters, the therapeutic properties of the Rivanazzano waters might too be related to their combined mineral and microbiological composition.

The general bacterial composition was not homogeneous at the different sites of collection. A relevant change in the native non-pathogenic microflora, although not statistically significant, was appreciated in both waters after the delivery from the natural sources to the spa resort. This could indicate that the bacterial phyla displayed a variable individual decreasing or increasing trend in both waters across the different sites of collection. Furthermore, some phyla completely disappeared, and some new ones appeared, after the water delivery from the natural sources to the spa resort and/or the heating at 70 °C.

In the Rivanazzano salso-bromo-jodic water, samples collected at the natural source tended to display a higher prevalence of bacteria from the *Sulfurimonadaceae*, the the *Arcobacteriaceae*, the *Desulfurivibrionaceae*, and the *Halothiobacillaceae* families.

The *Sulfurimonadaceae* and the *Arcobacteriaceae* families are from the *Campylobacteria* class (*Campylobacterota* phylum). *Sulfurimonadaceae* are known to be involved in the process of reducing nitrates, oxidising both sulphur and hydrogen, and they are coupled to autotrophy [37]. They are characterised by the ability to survive in extreme conditions and hypoxic environments, including deep-sea hydrothermal vents. The *Arcobacteriaceae* family includes non-pathogenic bacteria capable of forming dense solid sulphur-filament mats. These mats serve as essential substrates around hydrothermal vents, facilitating the colonization and settlement of various organisms by providing a stable habitat and attracting diverse marine life [38].

*Desulfovibrionaceae* is a family within the order *Desulfovibrionales*. They are all strictly anaerobic, mesophilic or moderately psychrophilic; most species are chemo-organoheterotrophic, but some others can be chemolithoheterotrophic. They are found in freshwater, brackish water and marine sediments, but also in biofilms, soil, sewage sludge, and animals [39].

The *Halothiobacillaceae* are a family of halotolerant, mesophilic, and obligate chemolithoautotrophic organisms. As halotolerant organisms, they tend to live in areas such as hypersaline lakes, coastal dunes, saline deserts, salt marshes, and inland salt seas and springs [40,41].

In the Rivanazzano sulphurous water collected at the natural source; the highest bacterial prevalence measured was from the *Sulfurovaceae*, *Desulfocapsaceae* and *Halothiobacillaceae* families (this was also true of *Halothiobacillaceae* in the salso-bromo-jodic water).

The family *Sulfurovaceae* of the *Campylobacterota* phylum [42] can be found in various habitats, including mangroves [43], nearshore sediments [44], shallow hydrothermal zones [45], glaciers [46], seagrass beds [47], and deep-sea hydrothermal vents [48,49,50].

The *Desulfocapsaceae* are a strict anaerobe family of *Thermodesulfobacteriota*. The family is considered one of the major sulphate-reducing bacterial groups in anoxic marine sediments, thereby accounting for one of the most important metabolic processes of anaerobic degradation of organic matter in the sea [51,52]. Interestingly, in both waters, all of these relevant bacterial families share an active biological role in chemo-auto-trophic processes [53]. Indeed, they belong to the most ancient bacterial populations since the origin of life [54].

We believe that these results are intriguing and require further rigorous investigation to explore the potential correlation between the waters’ therapeutic effects and their native non-pathogenic bacterial communities. In our opinion, the next step in this research line might be the lysate extraction from these selected bacterial families and their assessment on in vitro and ex vivo experimental cell models to investigate their potential favourable properties.

## 5. Limitations of the Study

This study represents an initial exploration of the microbiota present in the mineral waters of Rivanazzano; therefore, it was limited to the characterization of bacterial communities. A more comprehensive analysis would also include Archaea and Fungi. Future studies are desirable to extend these preliminary findings by comprehensively investigating the Rivanazzano waters’ microbiota.

Additionally, the sampling period was restricted to a three-month period (May to July), which did not adequately capture the potential seasonal fluctuations in bacterial community composition. Nevertheless, given the preliminary nature of this study, samples were collected during the peak working time of the spa resort to provide a relevant starting point for investigating potential correlations between microbial profiles and the therapeutic properties of the waters.

## 6. Conclusions

The Rivanazzano mineral waters revealed a rich and distinctive bacterial composition specific to the geographical area. It is reasonable to suggest that these bacterial communities, along with other microorganisms, may play a key role in the therapeutic effects of these waters, due to their ability to interact with the skin microbiota [6]. Actually, there is growing evidence that the skin microbiota is actively involved in the regulation of various diseases, as well as in wound healing, which is archetypal of all regenerative processes [55,56,57].

Reflecting on these considerations, a novel classification of natural mineral waters based on both their physicochemical characteristics and specific microbiota might be proposed.

## Figures and Tables

**Table 1:** Physical–chemical composition of the Rivanazzano salso-bromo-jodic and sulphurous mineral waters.

Parameter	Salso-Bromo-Jodic	Sulphurous
Temperature at the spring	13.9 °C	13.2 °C
pH	7.7	7.4
Conductivity at 20 °C	8100 μS/cm	1805 μS/cm
Fixed residue at 180 °C	12,400 mg/L	1334 mg/L
Oxidability	48.0 mg/L (O_2_)	39.0 mg/L (O_2_)
Silica (SiO_2_)	50.5 mg/L	19.9 mg/L
Bicarbonates (HCO_3_)	814 mg/L	705 mg/L
Chlorides (Cl)	7300 mg/L	192 mg/L
Sulphates (SO_4_)	35.7 mg/L	283 mg/L
Sodium (Na)	4490 mg/L	379.1 mg/L
Potassium (K)	24.5 mg/L	4.7 mg/L
Calcium (Ca)	143 mg/L	75 mg/L
Magnesium (Mg)	78.5 mg/L	31.2 mg/L
Dissolved iron (Fe)	3.37 mg/L	<0.01 mg/L
Ammonium ion (NH_4_)	22.4 mg/L	3.15 mg/L
Total phosphorus (P)	0.13 mg/L	<0.02 mg/L
Sulphide level (H_2_S)	not detectable	11.9 mg/L
Strontium (Sr)	15.8 mg/L	1.964 mg/L
Lithium (Li)	0.417 mg/L	0.066 mg/L
Aluminum (Al)	<0.01 mg/L	<0.01 mg/L
Bromides (Br)	37.1 mg/L	1.0 mg/L
Iodides (I)	13.9 mg/L	0.5 mg/L
Antimony (Sb)	<0.0005 mg/L	<0.0005 mg/L
Arsenic (As)	0.001 mg/L	<0.001 mg/L
Barium (Ba)	3.6 mg/L	<0.1 mg/L
Boron (B)	54.1 mg/L	3.24 mg/L
Cadmium (Cd)	<0.0001 mg/L	<0.0001 mg/L
Chromium (Cr)	0.001 mg/L	<0.001 mg/L
Copper (Cu)	<0.1 mg/L	<0.1 mg/L
Cyanides (CN)	<0.001 mg/L	<0.001 mg/L
Fluorides (F)	1.4 mg/L	0.26 mg/L
Lead (Pb)	<0.001 mg/L	<0.001 mg/L
Manganese (Mn)	0.06 mg/L	0.041 mg/L
Mercury (Hg)	<0.0001 mg/L	<0.0001 mg/L
Nickel (Ni)	<0.001 mg/L	0.001 mg/L
Nitrates (NO_3_)	<1.0 mg/L	<1.0 mg/L
Nitrites (NO_2_)	<0.002 mg/L	0.007 mg/L
Selenium (Se)	0.005 mg/L	<0.001 mg/L
Anionic surfactant agents	<50 μg/L	<50 μg/L
Mineral oils—dissolved or emulsified hydrocarbons	41 μg/L	34 μg/L
Benzene	<0.1 μg/L	<0.1 μg/L
Benzo[a]pyrene	not detectable	<0.003 μg/L
Benzo[b]fluoranthene	not detectable	<0.003 μg/L
Benzo[k]fluoranthene	not detectable	<0.003 μg/L
Benzo[ghi]perylene	not detectable	<0.003 μg/L
Dibenz[a,h]anthracene	not detectable	<0.003 μg/L
Indeno[1,2,3-cd]pyrene	not detectable	<0.003 μg/L
Acenaphthene	not detectable	<0.003 μg/L
Fluorene	not detectable	<0.003 μg/L
Phenanthrene	not detectable	<0.003 μg/L
Anthracene	not detectable	<0.003 μg/L
Fluoranthene	not detectable	<0.003 μg/L
Pyrene	not detectable	<0.003 μg/L
Benzo[a]anthracene	not detectable	<0.003 μg/L
Chrysene	not detectable	<0.003 μg/L
Chloroform	not detectable	<0.1 μg/L
Dibromochloromethane	not detectable	<0.1 μg/L
Bromodichloromethane	not detectable	<0.1 μg/L
Bromoform	not detectable	<0.1 μg/L
Trichloroethylene	not detectable	<0.1 μg/L
Tetrachloroethylene	not detectable	<0.1 μg/L
1,2-Dichloroethane	not detectable	<0.1 μg/L
1,1-Dichloroethylene	not detectable	<0.1 μg/L
1,2-Dichloroethylene	not detectable	<0.1 μg/L
1,1,1-Trichloroethane	not detectable	<0.1 μg/L
Carbon tetrachloride	not detectable	<0.1 μg/L
Total hardness	59.6 °F	31.6 °F

## Data Availability

The original contributions presented in the study are included in the article, further inquiries can be directed to the corresponding authors.
